# Impact of In-house *Candida auris* Polymerase Chain Reaction Screening on Admission on the Incidence Rates of Surveillance and Blood Cultures With *C. auris* and Associated Cost Savings

**DOI:** 10.1093/ofid/ofad567

**Published:** 2023-11-10

**Authors:** Rossana Rosa, Adriana Jimenez, David Andrews, Huy Dinh, Katiuska Parra, Octavio Martinez, Lilian M Abbo

**Affiliations:** Department of Infection Prevention, Jackson Health System, Miami, Florida, USA; Department of Infection Prevention, Jackson Health System, Miami, Florida, USA; Department of Epidemiology, Robert Stempel College of Public Health and Social Work, Florida International University, Miami, Florida, USA; Department of Pathology and Laboratory Medicine, University of Miami Miller School of Medicine, Miami, Florida, USA; Microbiology section, Department of Pathology, Jackson Memorial Hospital, Miami, Florida, USA; Microbiology section, Department of Pathology, Jackson Memorial Hospital, Miami, Florida, USA; Department of Pathology and Laboratory Medicine, University of Miami Miller School of Medicine, Miami, Florida, USA; Microbiology section, Department of Pathology, Jackson Memorial Hospital, Miami, Florida, USA; Department of Infection Prevention, Jackson Health System, Miami, Florida, USA; Division of Infectious Diseases, Department of Medicine, University of Miami Miller School of Medicine, Miami, Florida, USA

**Keywords:** PCR, *Candida auris*, fungemia, infection prevention, surveillance

## Abstract

**Background:**

The impact of strategies for rapid diagnostic screening of *Candida auris* on hospital operations has not been previously characterized. We describe the implementation of in-house polymerase chain reaction (PCR) testing on admission for screening of colonization with *C. auris,* associated process improvements, and financial impact.

**Methods:**

This study was conducted across an integrated health system. Patients were tested based on risk factors for *C. auris* carriage. Pre-intervention, the PCR was sent out to a reference laboratory, and postintervention was performed in-house. Changes in the incidence rates (IRs) of *C. auris* present on admission (CA-POA) and *C. auris* hospital-onset fungemia (CA-HOF) were assessed using interrupted time series analysis. The economic impact on isolation and testing costs was calculated.

**Results:**

Postintervention, the IR of CA-POA doubled (IRR, 2.57; 95% CI, 1.16–5.69; *P* = .02) compared with the pre-intervention period. The baseline rate of CA-HOF was increasing monthly by 14% (95% CI, 1.05–1.24; *P* = .002) pre-intervention, while during the postintervention period there was a change in slope with a monthly decrease in IR of 13% (95% CI, 0.80–0.99; *P* = .02). The median turnaround time (TAT) of the results (interquartile range) was reduced from 11 (8–14) days to 2 (1–3) days. Savings were estimated to be between $772 513.10 and $3 730 480.26.

**Conclusions:**

By performing in-house PCR for screening of *C. auris* colonization on admission, we found a doubling of CA-POA rates, a subsequent decrease in CA-HOF rates, reduced TAT for PCR results, and more efficient use of infection control measures. In-house testing was cost-effective in a setting of relatively high prevalence among individuals with known risk factors.

## Teaser

Following implementation of in-house PCR screening of *Candida auris* colonization on admission we observed changes in the incidence rates of *C. auris* present on admission and hospital-onset fungemia, while allowing for a more efficient use of infection control measures.

*Candida auris* is a multidrug-resistant fungus that has rapidly spread worldwide [1]. In the United States, *C. auris* was first reported in 2016 [2], and since then the percentage of cases reported each year has increased at a rapid pace, with a 95% and 200% increase in clinical and surveillance cases, respectively, seen in 2021 [3].

In the United States, patient colonization with *C. auris* and ongoing transmission have been mostly reported in patients residing in long-term acute care hospitals (LTACHs) and ventilator-capable skilled nursing facilities (vSNFs) [4–6]. Knowledge of a patient's *C. auris* colonization status on admission to a facility can help guide infection prevention activities [7], patient workflow, length of stay, and hospital discharges. Surveillance strategies for *C. auris* have been usually implemented in hospital units experiencing outbreaks, in locations considered as high risk for acquisition of *C. auris,* or among patients considered at risk for development of invasive disease [8–11]. Here, we report on the impact that performing surveillance for *C. auris* upon hospital admission had on our incidence rates of (1) *C. auris* present on admission (CA-POA) and (2) *C. auris* hospital-onset fungemia (CA-HOF), and we also present a cost-savings analysis on the use of in-house testing to guide infection prevention practices.

## METHODS

This study was conducted at 4 hospitals that are part of an integrated health system in Miami, Florida, United States, comprising nearly 2500 licensed beds. The pre-intervention period was from August 1, 2019, to July 31, 2021, and the postintervention period was from August 1, 2021, to January 31, 2023.

### Patient Identification and Testing

During the pre-intervention period starting August 1, 2019, all patients admitted to our hospitals were screened for *C. auris* using a 2-step process. The first step consisted of a questionnaire applied by nursing staff within the first 24 hours of admission with the aim of identifying patients at risk for colonization with *C. auris* (Supplementary Figure 1). The risk factors included in the questionnaire were arrival from a health care facility with known cases of *C. auris,* known history of colonization with *C. auris* or any carbapanemase-producing organism, presence of tracheostomy or mechanical ventilation, and history of hospitalization outside of the United States in the previous 12 months. This questionnaire was made part of the routine admission form on the electronic medical record (EMR). Patients with at least 1 risk factor for *C. auris* identified in the questionnaire were then placed on contact precautions. The second step consisted of screening for skin colonization with *C. auris* using a polymerase chain reaction (PCR) test. Samples were collected according to the Centers for Disease Control and Prevention (CDC) guidance from a composite of axilla and groin using a dry cotton swab. Patients would be identified from isolation lists by Infection Prevention (IP) staff, and sample collection for PCR would be coordinated between IP and nursing staff and sent for processing once a week per hospital to the CDC's Antimicrobial Resistance Laboratory Network (ARLN) in Tennessee, with a total of 2 send-out shipments per week for the health system.

Starting in August 2021 (postintervention period), patients continued to be screened with a 2-step process using the same EMR-based questionnaire. Patients identified as having at least 1 risk factor for colonization with *C. auris* had automatically generated orders for contact precautions and *C. auris* PCR testing. The PCR was performed in-house at the largest hospital of the system, and the 3 community hospitals sent their samples to this centralized location. Samples were processed daily Monday to Friday, and results were automatically reported on the electronic medical record, as well as sent to the IP service via email.

### Validation of the In-house *C. auris* PCR Platform

There are currently no available PCR tests for detection of *C. auris* colonization approved by the US Food and Drug Administration (FDA). Therefore, we developed and validated a commercially available in-house PCR test for use on a composite of axilla and groin samples. Analytical validation of the Diasorin analyte-specific reagent (ASR) *C. auris* reagents for the Liasion MDX instrument was performed by establishing the limit of detection (LOD), reproducibility, analytical sensitivity, and analytical specificity. The LOD was determined using isolates of *C. auris* Z485 from the Zeptometrix Panel with an original concentration of 3.22 × 10^9^ CFU/mL and extracting and testing 10-fold serial dilutions of the fungal solution. A confirmation of the LOD was determined using 20 extracted replicates. The LOD was determined as the lowest concentration where ≥95% of the replicates were detected and was estimated at 600 CFU/mL, with a mean cycle threshold (Ct) value of 32.97.

Analytical specificity of the *C*. *auris* laboratory-developed test (LDT) was evaluated using a panel of 12 isolated specimens positive for a pathogen other than *C. auris* by culture-based gold standard. The pathogens included were *C. albicans, C. glabrata, Aspergillus* spp., *Cryptococcus neoformans, Staphylococcus epidermidis, Escherichia coli, C. parapsilosis, C. haemolyticus, Saccharomyces cerevisiae, C. lusitaniae, C. kruseii,* and *C. duobushaemuloinii,* and the analytical specificity was 100% (all results were negative). Extensive analytical sensitivity and specificity studies are described by the manufacturer, including reactivity with several known, distinct *C. auris* clades. The assay primer pairs are directed toward a conserved region of the ITS2 spacer region of the rRNA gene. However, additional in silico evaluation of the molecular primers and probes was not possible because the specific sequences used for the assay are proprietary property of the manufacturer (Diasorin, Inc.). External laboratory correlations were determined using clinical specimens (n = 48) consisting of composites of axilla/groin swabs, collected in duplicate, with 1 batch sent to the CDC ARLN laboratory and the other processed using the Liasion MDX LDT. Using the ARLN results as the reference, the analytical sensitivity and specificity were estimated at 95.6% (22/23) and 100% (25/25), respectively.

### Infection Prevention Procedures

During the entire study period, isolation precautions consisted of universal gown and glove use for contact with the patient or their environment, single room placement, dedicated medical equipment, and cohorting of nursing staff when possible. If a patient was found to be *C. auris* PCR–positive, precautions were continued for the duration of admission. If a patient tested negative, they were not routinely retested, unless they were deemed newly exposed to *C. auris* during their hospital stay, or if requested by a receiving facility at discharge.

### Outcomes

For the purposes of this study, our primary outcome was to determine the impact of implementing in-house PCR testing on 2 outcomes: rates of CA-POA per 10 000 patient-admissions and rates of CA-HOF per 10 000 patient-days. As a secondary outcome, we sought to estimate the cost savings of performing the *C. auris* PCR in house vs sending it out to a reference laboratory.

### Statistical and Cost-Savings Analysis

An interrupted time series analysis (ITSA) with Poisson regression was used to assess the outcomes of interest, with prespecified models for the hypothesized changes [12]. For the CA-POA rates, a model assessing an immediate change in level of rates was developed, and for CA-HOF we used a model assessing for change in level and slope. Models were checked for autocorrelation and seasonality. Analysis was conducted on Stata, version 14.2.

Cost-savings were calculated by balancing the estimated costs of each isolation day with the cost of performing the *C. auris* PCR in-house vs sending out the test to the ARLN laboratory. The minimum and maximum costs of isolation were calculated using published estimates [13, 14]. The costs of in-house testing were inclusive of reagents and labor. All costs were converted to 2021 dollars using the US Bureau of Labor Statistics Consumer Price Index [15].

## RESULTS

### Testing Volumes

**Figure 1.**
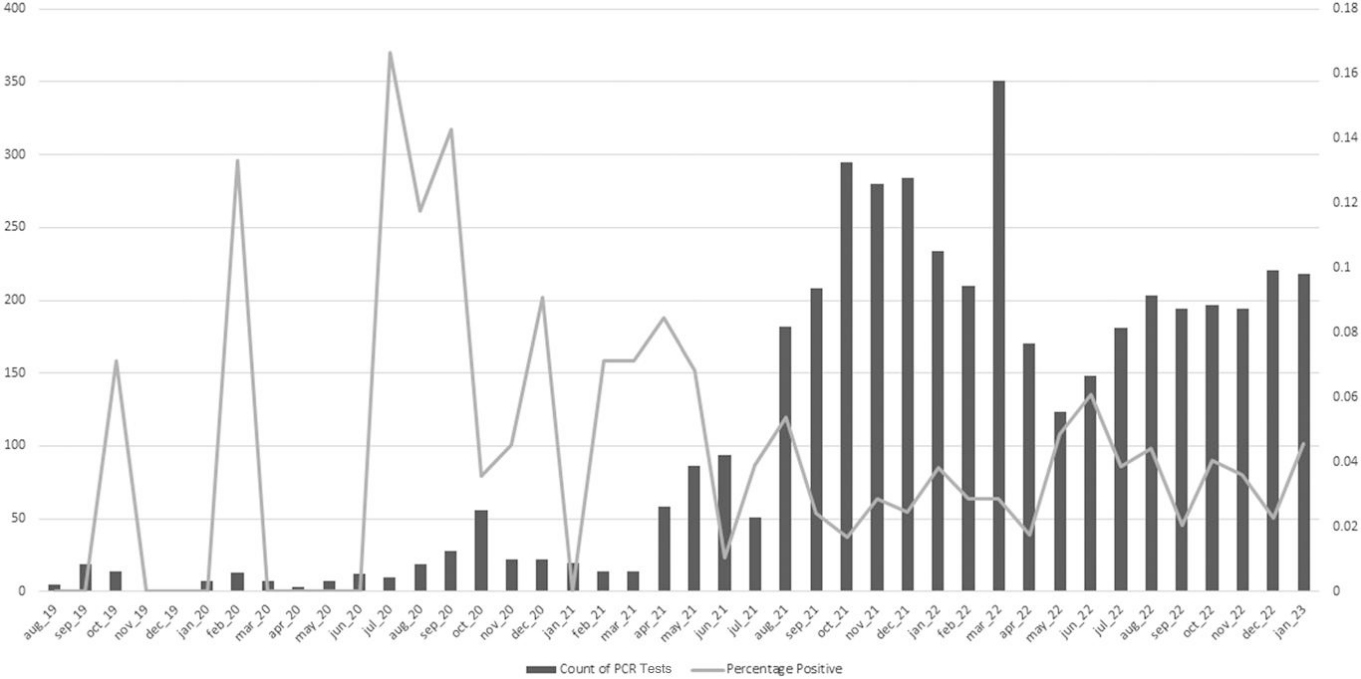
Monthly testing volume and percentage of PCR tests positive for *C. auris* present on admission. Abbreviation: PCR, polymerase chain reaction.

A total of 4478 colonization screening PCRs belonging to 4270 unique patients were performed during the study period. The median number of monthly tests during the pre-intervention period (interquartile range [IQR]) was 14.5 (7.5–26), and during the postintervention period it was 205.5 (181–234; *P* < .0001). The median percentage of positive tests (IQR) was 3.75% (0%–7.81%) during the pre-intervention period and 3.23% (2.40%–4.43%) during the postintervention period (*P* = .98). Testing volumes and percent positives are displayed in Figure 1.

### Rates of CA-POA

**Figure 2.**
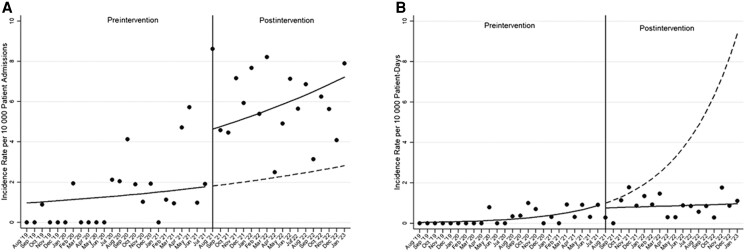
Impact of performing surveillance for *C. auris* colonization upon hospital admission on incidence rates of (*A*) *C. auris* present on admission per 10 000 patient-admissions and (*B*) *C. auris* hospital-onset fungemia per 10 000 patient-days. Circles represent observed rates. Solid lines represent estimated rates obtained from the interrupted time series model; segmented lines represent the counterfactual model without the effect of the intervention.

We identified 159 unique cases of CA-POA. Immediately before the onset of the intervention, the rate of CA-POA was 1.94 cases per 10 000 patient-admissions. The ITSA showed that during the pre-intervention period there was a baseline trend toward a monthly increase in the incidence rate of 3% (95% CI, 1.00%–1.06%; *P*  *=* .11), and following the intervention there was an immediate change, with an incidence rate ratio of 2.57 (95% CI, 1.16–5.69; *P* = .02) compared with the pre-intervention period (Figure 2*A*).

### Rates of CA-HOF

We identified 75 unique cases of CA-HOF. The first case of CA-HOF was identified at our institution in July 2019 (before our study period), with none were detected again until May 2020. Starting in November 2020, the BioFire BCID2 panel (which is capable of detecting *C. auris*) became available at our facilities. Immediately before the onset of the intervention, the CA-HOF rate was 0.91 cases per 10 000 patient-days. The ITSA showed that during the pre-intervention period, the baseline rate was increasing every month by 14% (95% CI, 1.05%–1.24%; *P*  *=* .002). No immediate change in rates was seen after introducing in-house testing, but throughout the postintervention period there was a change in slope showing a monthly decrease in rates of 13% (95% CI, 0.80%–0.99%; *P*  *=* .02) (Figure 2*B*).

### Cost-Savings Analysis

Patients screened for *C. auris* had a median length of stay (IQR) of 8 (4–15) days and remained on contact precautions as described above until *C. auris* PCR results were available. During the pre-intervention period, the median turnaround time (TAT) for the *C. auris* PCR (IQR) was 11 (8–14) days from admission to report, and during the postintervention period when PCR was performed in-house, the median TAT from admission to report (IQR) was 2 (1–3) days. Estimated isolation and testing costs are presented in Table 1. Considering that our positivity rate was 3%, we estimated that during the postintervention period the savings ranged from $772 513.10 to $3 730 480.26.

**Table 1.** Cost-Savings Analysis

	Pre-intervention (PCR Sent out to Refence Laboratory)^a^	Postintervention (PCR Performed In-house)
Microbiology laboratory turnaround time from admission to result report, median (IQR), d	11 (8–14)	2 (1–3)
Microbiology laboratory costs (reagents, equipment, and labor) per test, $	0	39.50
Startup costs,^b^ $		23 000.00
Duration of isolation precautions,^c^ d	8	2
Cost of isolation precautions per patient screened with a PCR-negative result, $	335.20–1379.68	123.30–384.42
Cost of isolating and testing 100 patients,^d^ $	33 520.00–137 968	13 084.20–41 546.28
Estimated cost savings during the postintervention period,^e^ $	772 513.10–3 730 480.26	

Abbreviations: ATCC, American Type Culture Collection; IQR, interquartile range; PCR, polymerase chain reaction.

^a^Samples sent to the Centers for Disease Control and Prevention Antimicrobial Resistance Laboratory Network.

^b^Startup costs including reagents, supplies, quality control materials, ATCC organisms, instrumentation, microbiology technologists’ and supervisor's time.

^c^Patients remained on contact precautions until *Candida auris* PCR results were reported back; median length of stay was 8 days.

^d^Not including startup costs; positivity rate 3%.

^e^Total number of surveillance tests performed during postintervention period: 3893; positivity rate: 3%.

## DISCUSSION

Performing in-house PCR testing for surveillance of *C. auris* colonization POA resulted in an increase in testing volumes and a more than doubling of rates of detected cases of CA-POA, and we subsequently observed a modest decrease in the rates of CA-HOF.

Our results indicate that there was already an underlying trend toward increase in the number of *C. auris* cases present on admission, which is consistent with national trends reported recently by Lyman et al. [3], and particularly in the state of Florida, which has the third highest number of cases reported in the United States [16]. Although our testing volumes had been increasing even when testing only twice per week and sending to a reference laboratory, performing in-house testing significantly allowed us to scale up our testing capabilities and detect more cases of *C. auris* present on admission and more promptly deploy our infection prevention strategies.

Although *C. auris* is a nationally notifiable disease of public health concern, there are no established mechanisms for systematic surveillance of this organism. Rowlands et al. [9] conducted a pilot study of universal *C. auris* screening on admission to 5 high-risk units in New York City (nursing homes and a hospital) and found that colonization percentages ranged from 3.6% to 22%, which is within the range of our findings. Furthermore, in the study by Rowlands et al., patients testing positive upon admission to the hospital were more likely to be intubated or have a tracheostomy present, have an indwelling device, or have received antimicrobials, highlighting the role of testing according to a patient's risk factors, which is the approach we undertook in our program.

Anecdotally, identification of *C. auris* status on admission can subsequently aid discharge planning (at least for those in a predicament similar to ours), whether by informing a long-term facility that one of their residents is already colonized with *C. auris* or by trying to find placement in a facility that will accept patients colonized with this particular multidrug-resistant organism.

In terms of the impact of in-house testing on CA-HOF, we hypothesize that through early identification of patients with *C. auris* colonization, infection prevention practices are promptly initiated, and there could also be a heightened awareness of the risk of transmission to other patients and of the risk of infection to the colonized patient.

Our report has several limitations. Although this intervention impacted 4 hospitals, they are part of an integrated health care system, with a central laboratory and an integrated Infection Prevention service, which may limit the generalizability of our strategy and findings. Furthermore, although *C. auris* is widespread in Southern Florida where we are located [16], the overall number of cases detected is relatively small, and therefore the utility of implementing this strategy in places with lower prevalence of *C. auris* is unknown. Furthermore, we are unable to report on the impact of in-house testing on intrahospital acquisition of *C. auris* (horizontal transmission), as we did not perform systematic surveillance of high-risk patients who initially tested negative. An expanded cost-savings analysis including impact on length of stay was not undertaken as patients colonized with *C. auris* often have complex comorbidities and their colonization status may not be the sole driver of the decision to discharge them.

In conclusion, by performing in-house PCR testing on admission for the detection of colonization with *C. auris*, we noted a more than doubling of cases of CA-POA, and we subsequently observed a lowering of the incidence rates of CA-HOF. In-house testing resulted in cost savings in our setting and could be considered in areas of high prevalence to prevent horizontal transmission and decrease invasive infection. Further studies are needed to more precisely outline surveillance strategies for the detection of this organism, both in acute and long-term care settings, and in accordance with local prevalence.

## Supplementary Material

ofad567_Supplementary_Data

## Supplementary Data

Supplementary materials are available at *Open Forum Infectious Diseases* online. Consisting of data provided by the authors to benefit the reader, the posted materials are not copyedited and are the sole responsibility of the authors, so questions or comments should be addressed to the corresponding author.
