# Supplementary figures and images for: Impact of In-house Candida auris Polymerase Chain Reaction Screening on Admission on the Incidence Rates of Surveillance and Blood Cultures With C. auris and Associated Cost Savings
Source: Open Forum Infect Dis. 2023 Nov 10;10(11):ofad567. doi: 10.1093/ofid/ofad567 (PMC10665036; doi:10.1093/ofid/ofad567)

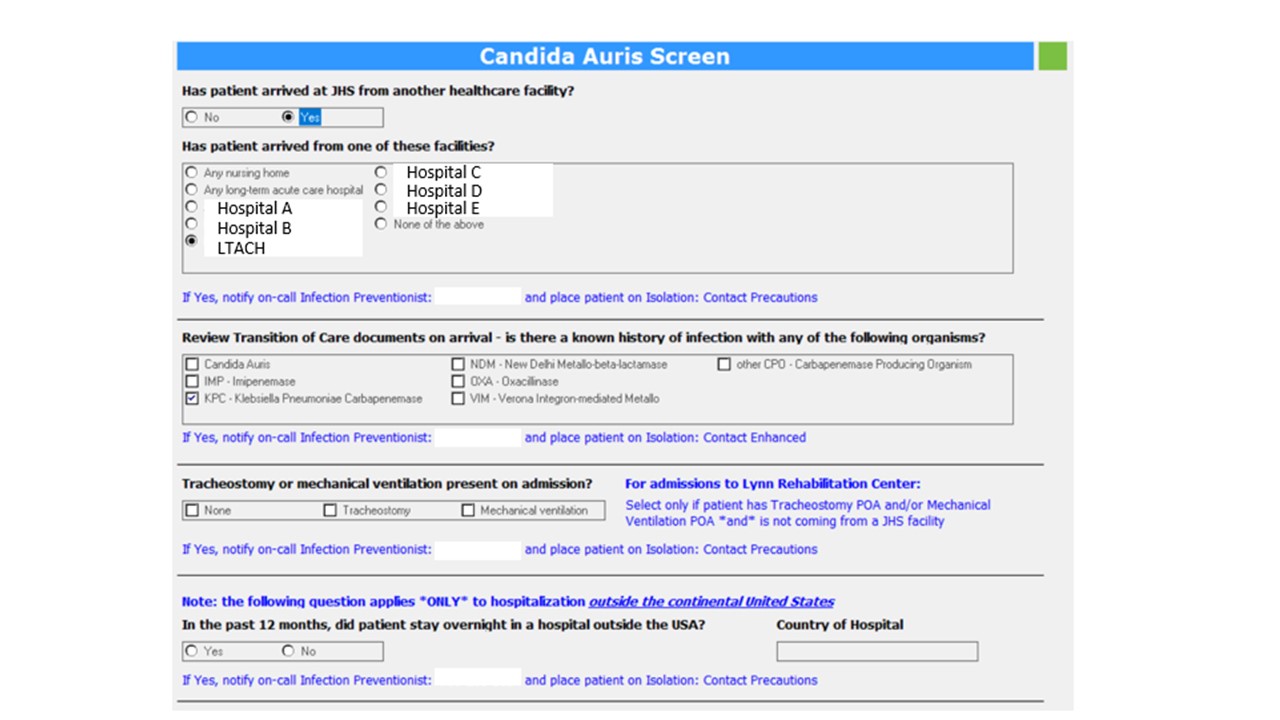

Supplement: ofad567_Supplementary_Data [file ofad567_supplementary_data.jpeg]
